# Intranasal administration with recombinant *Bacillus subtilis* induces strong mucosal immune responses against pseudorabies

**DOI:** 10.1186/s12934-019-1151-8

**Published:** 2019-06-07

**Authors:** Jialu Wang, Yongheng Wang, En Zhang, Mengyun Zhou, Jian Lin, Qian Yang

**Affiliations:** MOE Joint International Research Laboratory of Animal Health and Food Safety, College of Veterinary Medicine, Nanjing Agricultural University, Weigang 1, Nanjing, 210095 Jiangsu People’s Republic of China

**Keywords:** Pseudorabies virus (PRV), Recombinant *Bacillus subtilis*, Mucosal immunity, Vaccines

## Abstract

**Background:**

Pseudorabies caused by pseudorabies virus (PRV) mainly infects the swine and seriously threatens the biosafety of the other animals, including humans. Since 2011, the outbreaks of PRV mutants have caused enormous economic losses in the swine industry, and traditional vaccines cannot offer enough protection. PRV can transmit by direct contact, aerosol transmission and pollutants. PRV mainly transmit through the nasal mucosa. After infecting the nasal epithelial cells, PRV can quickly infect the olfactory nerve and establish a potential infection of sensory neurons. Therefore, nasal immunity can effectively prevent viral colonization infection. Recombinant *Bacillus subtilis* has been widely used to deliver antigen and achieve adequate protective immune responses.

**Results:**

The present study successfully constructed recombinant *Bacillus subtilis* (*B. subtilis*) expressing the dominant antigen regions of PRV gC and gD proteins (named *B. subtilis*-gCa and *B. subtilis*-gDa). Furtherly, we evaluated the immunogenicity of the two recombinant *B. subtilis* in mice. The mice intranasal administration with *B. subtilis*-gCa and *B. subtilis*-gDa effectively stimulated IgA and IgG immune responses, further regulated specific T lymphocytes proliferative response by IFN-γ and IL-10, and ultimately produced high titers of neutralizing antibodies against PRV infection. In particular, *B. subtilis*-gDa possessed more excellent immune effect than *B. subtilis*-gCa in mice.

**Conclusions:**

These results suggested that *B. subtilis*-gCa and *B. subtilis*-gDa could trigger high levels of mucosal and systemic immune responses and would be potential candidates for developing PRV vaccines.

**Electronic supplementary material:**

The online version of this article (10.1186/s12934-019-1151-8) contains supplementary material, which is available to authorized users.

## Introduction

*Bacillus subtilis (B. subtilis)* is a nonpathogenic Gram-positive probiotic and has been extensively used in humans and animals [1]. *B. subtilis* is a potential and cost-effective substitute of antibiotics and generally regarded as safe (GRAS) by food and drug administration (FDA) in American. Besides, *B. subtilis* plays a potential role in regulating cellular immunity and humoral immunity. Administration of *B. subtilis* can resist many diseases in animals, like porcine epidemic diarrhea (PED) [2], foot-and-mouth disease (FMD) and influenza [3, 4]. Furthermore, *B. subtilis* is also easy to be genetically manipulated and many molecular tools have been developed. Recent researches indicated that recombinant *B. subtilis* could elicit unique immune responses [5].

Pseudorabies (PR) is a serious veterinary pathogen that can end up with abortions in sows and mortality of piglets. PR is caused by the pseudorabies virus (PRV). PRV belongs to the herpesviridae family. PRV mainly infects the swine and always directly causes lethal infection, regardless of the age of the animal in other species. All kinds of vaccines play important roles to control PRV over the years [6]. However, since 2011, some new emerging PRV variants have swept many pig farms in China and conventional vaccines could not provide enough protection against the new PRV mutants [7]. The new PRV mutants severely hinder the development of the Chinese swine industry and threaten the world’s bio-security [8].

In addition, traditional PRV vaccines exist some problems of security, which furtherly lead to the spread of PRV across different species [9]. Recently, epidemiological data analysis indicated that PRV had the potential to infect humans [10]. The interspecies transmission mechanism and evolutionary dynamics of PRV also proved the potential risk of PRV transmission between humans and animals [11]. Therefore, it is very urgent and meaningful to develop a more efficacious and secure vaccine to eradicate the virulent PRV variants.

The most common pathway of PRV infection is through nasal mucosa. When PRV transmits through the nasal cavity, the virus replicates in the upper respiratory tract before attacking sensory nerve endings, crossing synapses to infect neurons and invading the nervous system [12]. Therefore, intranasal immunization might be an ideal measure against the PRV infection.

Intranasal administration is an effective and attractive route for immunization against diseases caused by upper respiratory tract infections of pathogens [13]. Moreover, it does not require needles and syringes, and can be easily applied to large-scale immunization [14]. Intranasal administration is able to simulate the natural infection of pathogens and induces immunoglobulin A (IgA) production in the nasal mucosa [15]. The unique structure (easily accessible and highly vascularized) of the nasal cavity provides a favorable immunological environment which contains abundant T and B cells, dendritic cells, macrophages and lymphoid tissues such as nasal-associated lymphoid tissue (NALT) [16]. Nasal administration can induce both mucosal and systemic immune responses to defend the nasal from the entry of pathogens [17]. Therefore, intranasal vaccination has great potential in clinical use [18].

Although intranasal administration has many promising advantages, some problems still exist to limit its development. Firstly, a variety of enzymes always degrade the antigens carried by the vaccines in the nasal cavity [19]. Secondly, rapidly oscillating cilia greatly shorten the intranasal residence time of vaccines and reduce the antigens uptake, which result in the vaccines failure [20]. At last, the complex and close-knit geometry of the nasal cavity challenges the vaccines to deliver antigens to mucosal surfaces high and deep [21]. Therefore, nasal immunization usually requires the use of vaccine adjuvants or delivery systems to protect the antigen immunogenicity and to increase antigen delivery efficiency.

In the present study, we constructed a recombinant *B. subtilis* that expressed the major antigenic region of PRV gC or gD proteins and evaluated the immune effect of the recombinant *B. subtilis*. Our results indicated that the recombinant *B. subtilis* was beneficial to the mucosal immune system development and could effectively generate specific antibodies against PRV infection, which suggests a potential approach for preventing PRV infection.

## Materials and methods

### Virus, bacterium, plasmids and cell lines

PRV strain ZJ01 was kindly provided by Professor Ping Jiang (Nanjing Agricultural University) [22]. The viruses were propagated in the porcine kidney cell line (PK-15) and cultured by DMEM (Life Technologies, Shanghai, China) supplemented with 2.5% fetal bovine serum (FBS, Life Technologies) at 37 °C with 5% CO_2_ in a humidified incubator. The viruses were a UV-inactivated at UV dose of 1000 mJ/cm^2^ to achieve a complete loss of infectivity [23]. The *B. subtilis* WB800 was kindly provided by Dr. Xuewen Gao (from the department of plant pathology, Nanjing Agricultural University) [24]. The plasmid p43NMK was stored in our lab and constructed by Dr. Xiaozhou Zhang (from the department of Microbiology, Nanjing Agricultural University) [25]. The plasmid pLJM1-EGFP was a gift from David Sabatini (Addgene plasmid # 19319; http://n2t.net/addgene:19319; RRID: Addgene_19319) [26].

### Construction of recombinant *B. subtilis*

**Fig. 1:**
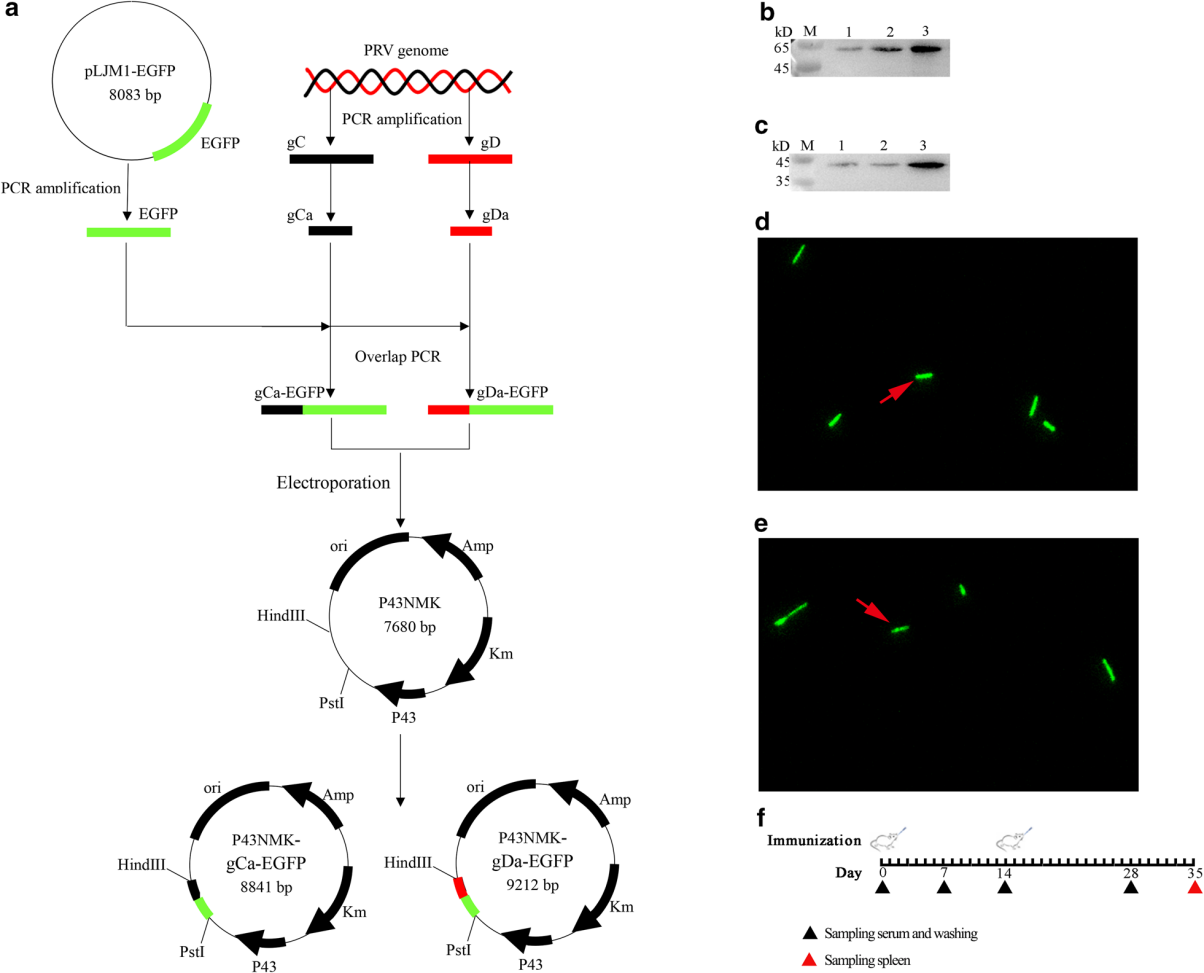
The constitution of recombinant *B. subtilis*-gCa and *B. subtilis*-gDa. **a** The recombinant *B. subtilis*-gCa and *B. subtilis*-gDa were constructed by common molecular biology technique. The PRV gCa, gDa, EGFP gene were connected by overlap PCR, then the specific connected fragments were inserted into the linearized plasmid p43NMK by in-fusion clone to get p43NMK-gCa-EGFP and p43NMK-gDa-EGFP. Furthermore, the two recombinant plasmids were transformed into *B. subtilis* WB800 by electroporation. The fusion proteins of *B. subtilis*-gDa (**b**) and *B. subtilis*-gCa (**c**) were detected by western blotting in 24 h, 48 h and 60 h. Besides, the green fluorescence were observed the *B. subtilis*-gCa (**e**) and *B. subtilis*-gDa (**d**) by confocal microscope. **f** The schematic of the immunization, the intranasal administration was performed at 0 and 14 day. In addition, the black triangle (under the line) indicated the time point of sampling the washings (including BAL and vagina) and serum, the red triangle (under the line) represented the last sampling and sampled the washings, serum and spleen

PRV strain ZJ01 viral DNA was extracted as described previously by using Viral DNA kit (Omega Bio-Tek Inc., Norcross, GA, USA) [27]. Then, we amplified the gC and gD gene by using the viral genome as the template (Table 1, Additional file 1: Figure S1E). Next, we analyzed the dominant antigen regions of these two proteins by TMHMM 2.0 and DNAstar (Additional file 1: Figure S1A–D). The amplified dominant antigen regions were named gCa and gDa (Additional file 1: Figure S1F). In addition, we amplified the EGFP gene by using the plasmid pLJM1-EGFP as the template (Table 1) (Additional file 2: Figure S2A). Next, we connected the gCa and gDa with the EGFP by overlap extension PCR to obtain gCa-EGFP and gDa-EGFP fragments (Additional file 2: Figure S2B, C). Afterwards, these two fragments were inserted into the plasmid p43NMK which was linearized by PstI and HindIII (Takara, Kyoto, Japan) to construct recombinant plasmid p43NMK-gCa-EGFP and p43NMK-gDa-EGFP. At last, the two recombinant plasmids were transformed into *B. subtilis* WB800 by electroporation using the Eppendorf Multiporator (Eppendorf AG, Hamburg, Germany) as previously described (Additional file 3: Figure S3) [28]. The recombinant *B. subtilis* WB800 strains were named *B. subtilis*-gCa and *B. subtilis*-gDa (Fig. 1a).

**Table 1:** The genetic information

Gene name	Gene length (bp)	Template	Primer name	Sequence (5′–3′)
gC	1386	PRV genome	P1	TGCTGGCGCTCTACACGG
			P2	GCACGATGGCTAGGATGGC
gD	1040	PRV genome	P3	CCGTACACCGAGTCGTGGCA
			P4	CCCCTCAGGCGGAAGAAGATG
gCa	444	gC	P5	ACGACGGCGCTCGGCACG
			P6	TCGCCCTTGCTCACCATGCTGAAGAGGAGCCGC
gDa	815	gD	P7	GTCCCCTCGCCCTTCG
			P8	CGCCCTTGCTCACCATGTCTCGGGCCTCGGGG
EGFP (Ca)	717	pLJM1-EGFP	P9	GCGGCTCCTCTTCAGCATGGTGAGCAAGGGCGA
			P10	CTTGTACAGCTCGTCCATG
EGFP (Da)	717	pLJM1-EGFP	P11	GCGCCGCACCACGCCGATGGTGAGCAAGGGCG
			P12	CTTGTACAGCTCGTCCATG
gCa-EGFP	1161	gCa, EGFP	P13	TTGTAACACATGCCTCAGCTGCAGGAACGACGGCGCTCGGC
			P14	TGATTACGCCAAGCTTTTACTTGTACAGCTCGTCCATGCC
gDa-EGFP	1532	gDa, EGFP	P15	GTTTTTGTAACACATGCCTCAGCTGCAGGAGTCCCCTCGCCCTTCG
			P16	TGATTACGCCAAGCTTTTACTTGTACAGCTCGTCCATGCC

### Analysis of fusion proteins

*Bacillus subtilis*-gCa and *B. subtilis*-gDa were grown in 5 ml LB medium at 37 °C, 220 rpm for 24 h, 48 h and 60 h. Then, the two strains of bacteria were washed three times by sterile phosphate-buffered saline (PBS). After washed, these were centrifuged for 5 min at 12,000×*g* at 4 °C and re-suspended in 500 μl PBS. And these bacteria were disrupted to release the target proteins by Ultrasonic Cell Crusher (Xianou Tech, China) as previously described [29]. Furthermore, the proteins were detected by western blotting (Fig. 1b, c), in which the mouse anti-EGFP (Sigma) and HRP-conjugated goat anti-mouse IgG (Sigma) were used. Then the blots were developed by ECL enhanced chemiluminescence. The images were captured using a 4600SF Gel Image System (Tanon, China).

Besides, the re-suspended bacteria were observed under Zeiss LSM710 confocal microscope (Zeiss, Germany). The images were analyzed using ZEN 2012 (Carl Zeiss) (Fig. 1d, e).

### Animals and immunization programs

Sixty healthy BALB/c female mice (5-week-old, purchased from Experimental Animal Center of Yangzhou University) were randomly divided into four groups (n = 15/group) and housed under similar conditions in different cages (in order to avoid probiotic cross-contamination). The animal experiments was permitted by the Institutional Animal Care and Use Committee of Nanjing Agricultural University and followed the National Institutes of Health’s guidelines for the performance of animal experiments. Mice of groups 1–4 were intranasally administration with 20 μl of PBS (1), 10^9^ CFU WB800 in combination with 10^5^ TCID_50_ inactivated PRV (*B. subtilis*-PRV) (2), 10^9^ CFU *B. subtilis*-gCa (3), and *B. subtilis*-gDa (4). The immunization protocol was performed on day 0 and day 14 (Fig. 1f). The sera and washings (broncheoalveolar lavage (BAL) and vagina washings) were collected at day 0, 7, 14, 28, 35. At day 35, the last three mice of each group were sacrificed for lymphocyte proliferation assay and the analysis of CD3^+^, CD3^+^CD4^+^ and CD3^+^CD8^+^ T lymphocytes in the spleen.

### Detection of PRV specific antibodies by indirect ELISA

The indirect ELISA was performed as previously described protocol [30]. The inactivated PRV virus particles were coated on 96-well microliter plates at a concentration determined by preliminary experiments. The sera were incubated with the plates, and bound antibodies were detected with HRP-conjugated goat anti-mouse or goat anti-porcine antibody (Sigma). The plates were incubated with the color substrate 3,3,5,5-tetramethyl benzidine (TMB) (Sigma) at room temperature for 15 min for color development. The enzyme–substrate reaction was stopped by adding 1% SDS in each well. The optical density (OD) was read at 630 nm.

### Analysis of CD3^+^, CD3^+^CD4^+^ and CD3^+^CD8^+^ T lymphocytes in spleen

The spleen was removed from the mice, then cut into pieces and ground gently through a 200 mesh sterile nylon net. The cell suspension was carefully collected and laid on the RPMI Medium 1640 (Thermo Fisher Scientific). The separated spleen mononuclear cells were incubated with fluorochrome-conjugated antibodies directed at the following CD markers: PE anti-mouse CD3, FITC anti-mouse CD4, and PE-Cy7 anti-mouse CD8 (BD Biosciences Pharmingen). Gated CD3 positive events were analyzed for CD3^+^CD8^+^ and CD3^+^CD4^+^ T cells. Flow cytometry was performed using an FC500 flow cytometer (Beckman Coulter, Fullerton, CA) and analyzed using Beckman Coulter CXP software. Flow cytometry analysis was performed on BD Facscalibur (BD Biosciences) instruments and analyzed by FlowJo software [31].

### Lymphocyte proliferation assay

At 35 days, lymphocytes were isolated from the spleens as previously described. The cells were re-suspended at 5 × 10^6^ cells/ml with RPMI Medium 1640 (Thermo Fisher Scientific) supplemented with 10% FBS, and then transferred into 96-well plates (100 μl per well), followed by PBS, Con A, *B. subtilis*-PRV, *B. subtilis*-gCa, and *B. subtilis*-gDa. The plates were incubated for 72 h at 37 °C. Then, the proliferative responses were detected using the standard CCK8 (Solarbio, China) method as previously described [32].

### Real-time quantitative PCR

After total RNA was extracted from the lung and vagina, 500 ng of total RNA from each sample was reversely transcribed using the PrimeScript RT reagent kit (Takara, Japan). Real-time PCR was performed on cDNA using SYBR Premix Ex Taq (Takara, Japan) on the Line Gene 9600 Plus QPCR system (Bioer, China), using Line Gene 9600 Plus software for comparative quantification. All reactions were carried out in triplicate using a 25 μl volume. PCR amplification was carried out using an initial denaturation at 95 °C for 5 min, followed by 40 cycles for 30 s at 95 °C, 30 s at 60 °C, and 30 s at 72 °C. The primers for the genes are provided as follows: IFN-γ (F: TGGCATAGATGTGGAAGAA, R: GTGTGATTCAATCAATGACGCTTA), IL-10 (F: GGTTGCCAAGCCTTGTCTGA, R: AGGGAGTTCACATGCGCCT), GAPDH (F: GCCCAAGATGCCCTTCAGT, R: CCTTCCGTGTTCCTACCCC).

### Plaque reduction neutralization test (PRNT)

PRV neutralizing antibodies were measured in sera by PRNT. The test was performed as previously described with minor modifications [33]. The sera were inactivated at 56 °C for 30 min before used in the assay. 450 μl of sera were twofold serially diluted and mixed with 50 μl viral suspension containing 10^5^ TCID_50_ PRV virus for 1 h at 37 °C in 12-well flat bottomed tissue culture plates. The mixture was then inoculated for 1 h at 37 °C and 5% CO_2_. Then, the mixture was inoculated with PK-15 cells suspension (ca. 1.0 × 10^6^ ml^−1^) for another 3–4 days. After staining with Crystal Violet, the plates were observed under a microscope for cytopathic effect.

### Statistical analysis

Data were represented as the mean ± SD of three replicates per test in a single experiment. GraphPad Prism V6.0 (San Diego, CA, USA) was used to perform statistical analyses. Tukey’s multiple comparison tests and one-way analysis of variance (ANOVA) were used to analyze the significance of the difference between means. P-values less than 0.05 (*P* < 0.05) were considered significant and P-values less than 0.01 (*P* < 0.01) were considered as highly significant.

## Results

### Construction and validation of the recombinant *Bacillus subtilis*

We cloned PRV gCa, gDa, EGFP gene and constructed two expression plasmids (Fig. 1a) by common molecular biology technique. Then, we transformed the plasmids into the *B. subtilis* WB800 by electroporation (Additional file 3: Figure S3). Furthermore, we selected positive clones by kanamycin resistant screening. At last, we analyzed the fusion protein expression by western blotting in 24 h, 48 h and 60 h (Fig. 1b, c) and green fluorescence (Fig. 1d, e). The expression of gDa-EGFP fusion proteins increased over time and peaked in 60 h (Fig. 1b). The similar results were detected in gCa-EGFP fusion proteins (Fig. 1c). In addition, the expression of *B. subtilis*-gDa were higher than *B. subtilis*-gCa. Moreover, we could clearly observe the *B. subtilis*-gCa (Fig. 1e) and *B. subtilis*-gDa (Fig. 1d) with green fluorescence by confocal microscope. These results indicated that recombinant *Bacillus subtilis* (*B. subtilis*-gCa and *B. subtilis*-gDa) were successfully constructed.

### The specific antibody in mice induced by recombinant *Bacillus subtilis*

We detected the anti-PRV-specific secreted IgA (sIgA) and IgG antibodies by ELISA. The anti-PRV-specific IgG antibodies were detected in sera. The anti-PRV-specific secreted IgA antibodies were mainly detected in broncheoalveolar lavage (BAL) and vagina washings. The scheme of intranasal immunization and specimen collection is shown as Fig. 1f.

**Fig. 2:**
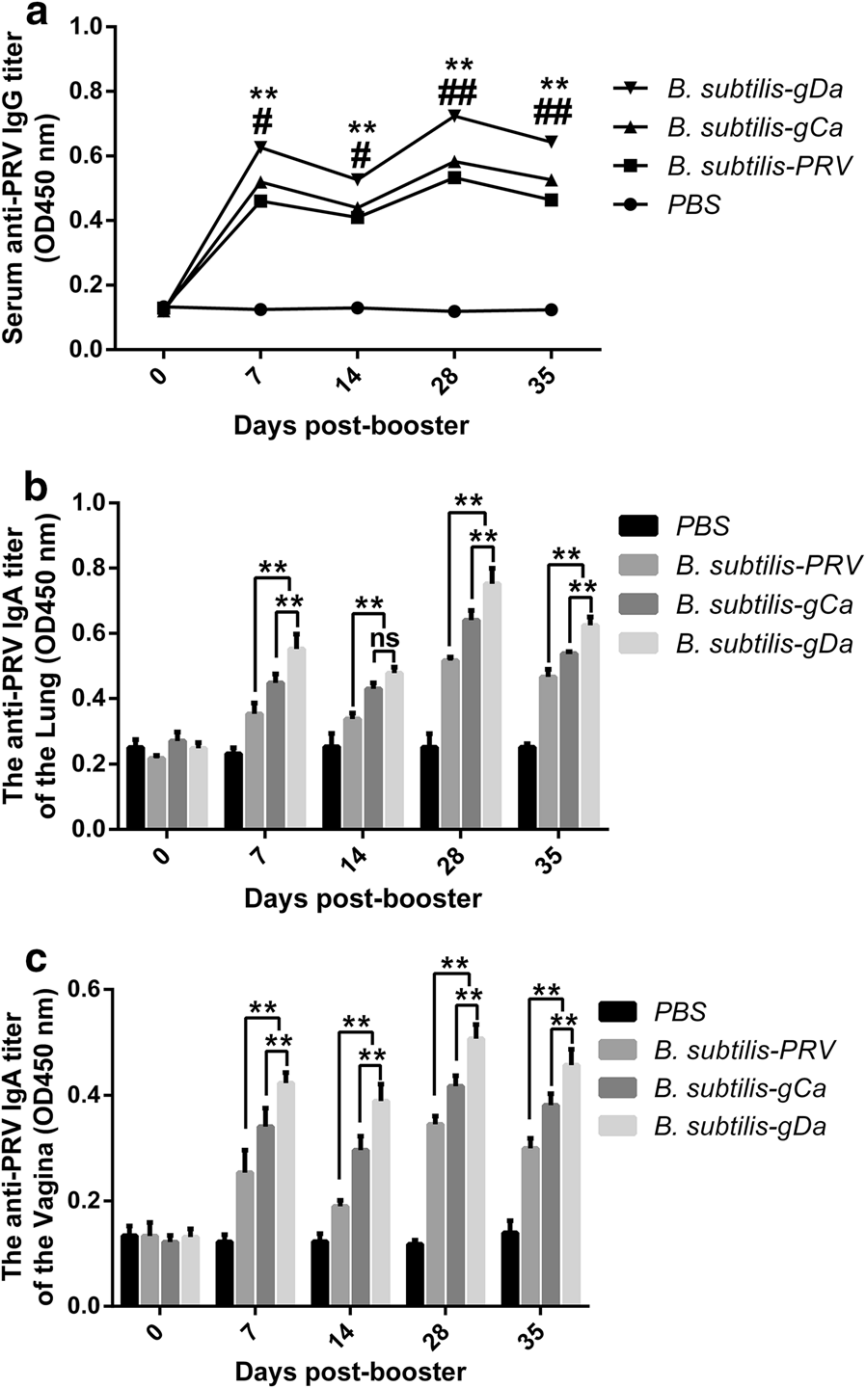
The specific antibody in mice induced by recombinant *B. subtilis*-gCa and *B. subtilis*-gDa. **a** Detection of anti-PRV IgG antibody in serum from immunized mice by indirect ELISA. *P < 0.05, **P < 0.01 compared to *B. subtilis*-PRV. ^#^*P* < 0.05, ^##^*P* < 0.01 compared to *B. subtilis*-gCa. **b** The anti-PRV specific mucosal SIgA antibody in broncheoalveolar lavage (BAL) by indirect ELISA. **P < 0.01. **c** The anti-PRV specific mucosal SIgA antibody in vagina by indirect ELISA. Data were expressed as the mean ± SD. **P < 0.01

The results indicated that the titers of specific IgG antibody in the serum of immuned mice increased by immunization times, and presented a gradual increase trend (Fig. 2a). After the boost immunization, the antibody reached the highest level. Besides, the group that undertook intranasal immunization with *B. subtilis*-gDa kept the highest levels of IgG than the other three groups. The groups that were immunization with *B. subtilis*-gCa and *B. subtilis*-PRV almost remained the same level that were clearly higher than the PBS group. These data showed that the recombinant *Bacillus subtilis* could effectively stimulate systemic immune responses.

Furthermore, the titers of mucosal anti-PRV-specific secreted IgA assessed the mucosal immune responses. As were shown in Fig. 2b, intranasal immunization with *B. subtilis*-gDa significantly induced a higher level of IgA in BAL on days 7, 28 and 35, compared with the other two groups (*P *< 0.01). In addition, the *B. subtilis*-gCa had the same comparative results compared with the *B. subtilis*-PRV group on four sampling points (*P *< 0.01). However, there was no significant difference between the *B. subtilis*-gDa and *B. subtilis*-gCa on days 14 (*P* > 0.05). Figure 2c showed the trend of sIgA levels in the vagina washings and the general trend was the same as BAL. Besides, it is worth noting that the titers of IgA in BAL were higher than vagina washings.

### The percentage of CD3^+^, CD3^+^CD4^+^, and CD3^+^CD8^+^ T lymphocytes in spleen

**Fig. 3:**
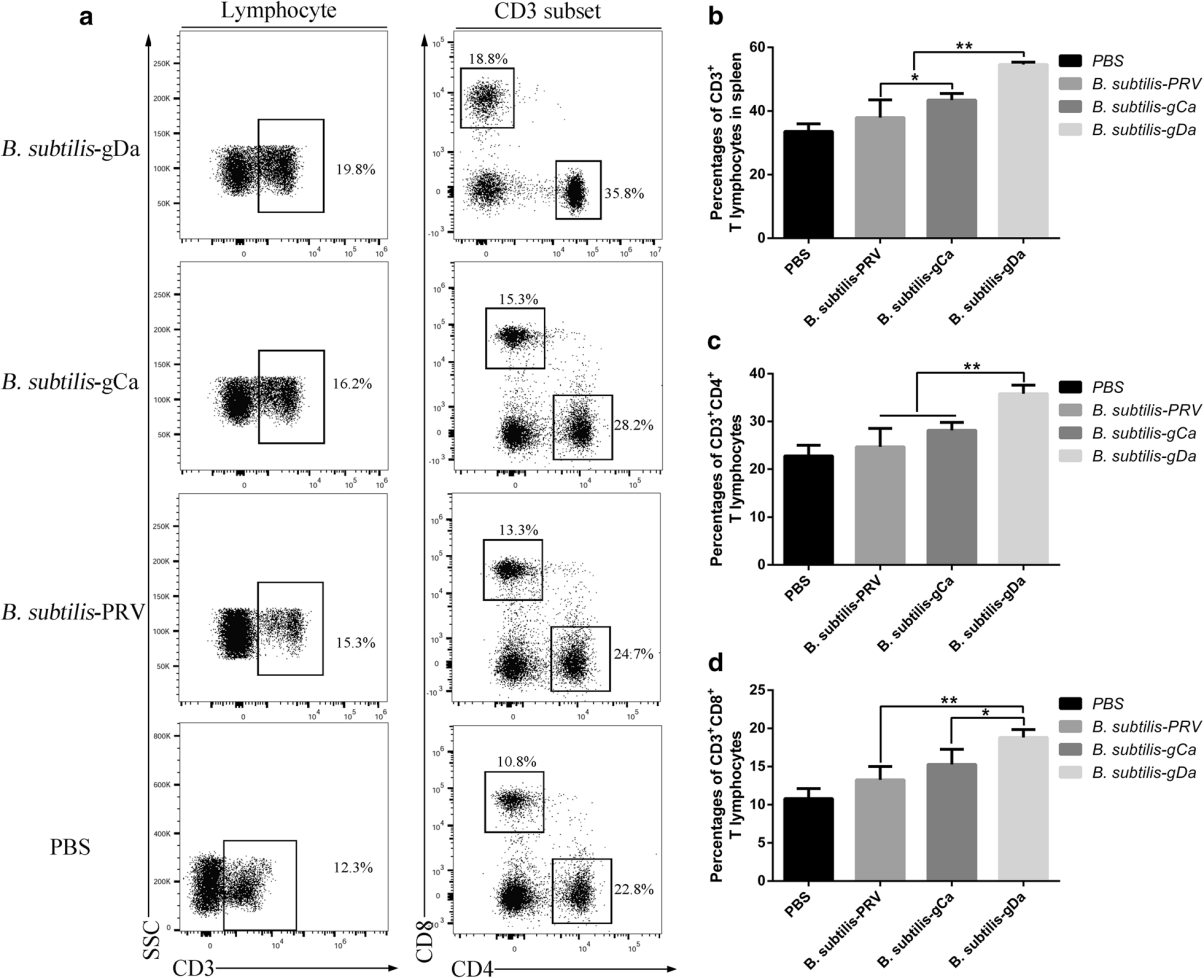
The percentage of CD3^+^, CD3^+^CD4^+^, and CD3^+^CD8^+^ T lymphocytes in spleen. The spleen was separated from the mice at 35 day. **a** The representative flow cytometry plots showing CD3^+^, CD3^+^CD4^+^, and CD3^+^CD8^+^ T lymphocytes in the spleen. The quantification of the frequencies were shown in** b** CD3^+^,** c** CD3^+^CD4^+^ and** d** CD3^+^CD8^+^ T cells. Data were expressed as the mean ± SD. * *P* < 0.05, ** *P* < 0.01

The percentage of CD3^+^, CD3^+^CD4^+^, and CD3^+^CD8^+^ T lymphocytes in spleen was an important indicator of the specific immunity in the body. Flow cytometry was performed to measure the changes in the percentage of CD3^+^, CD3^+^CD4^+^ and CD3^+^CD8^+^ T lymphocytes in spleen (Fig. 3a). After intranasal immunization with *B. subtilis*-gDa, as shown in Fig. 3b, the percentage of CD3^+^ T lymphocytes significantly increased than other groups (*P *< 0.01). Besides, the *B. subtilis*-gCa group was higher than *B. subtilis*-PRV group in CD3^+^ T lymphocytes (*P *< 0.05). Moreover, intranasal immunization with *B. subtilis*-gDa induced a higher percentage of CD3^+^CD4^+^ T lymphocytes than the other two groups (*P *< 0.01). However, there was no significant difference (*P* > 0.05) between the *B. subtilis*-gCa and *B. subtilis*-PRV group (Fig. 3c). Furthermore, the percentage of CD3^+^CD8^+^ T lymphocytes was similar with Fig. 3b (*P *< 0.01 or *P *< 0.05).

### The proliferation of lymphocytes in spleen

**Fig. 4:**
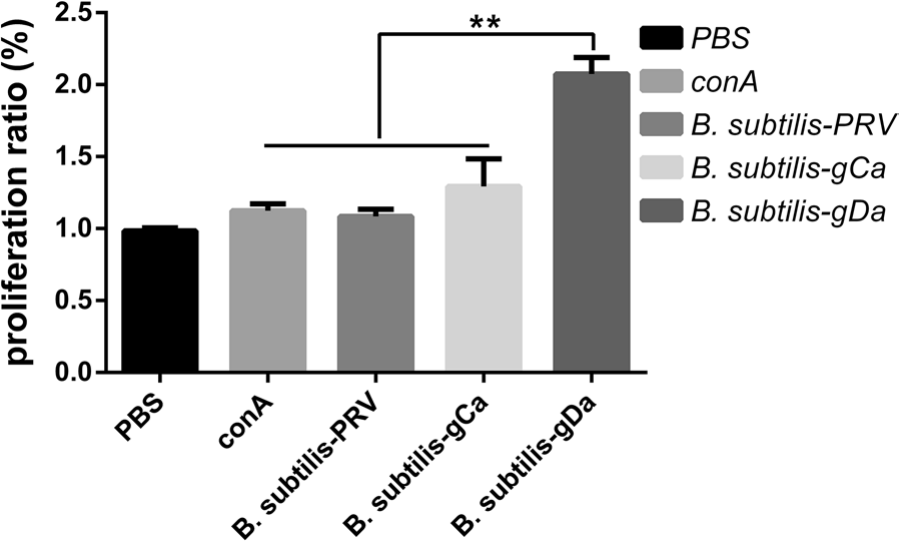
The proliferation of lymphocytes in spleen. Splenocytes were prepared at 35 day and cultured with PBS, Con A, *B. subtilis*-PRV, *B. subtilis*-gCa and *B. subtilis*-gDa for 72 h at 37 °C. Then, the proliferative responses detected by CCK8 method and shown as a proliferation ratio. Data were expressed as the mean ± SD. **P < 0.01

The proliferation of lymphocytes in spleen was analyzed by CCK8 assay. The data showed that *B. subtilis*-gDa significantly enhanced the proliferation of lymphocytes in spleen followed the trend shown in Fig. 4 (*P *< 0.01). And the *B. subtilis*-gCa and *B. subtilis*-PRV group remained at the same level as ConA group (*P *> 0.05).

### Determination of cytokine responses

**Fig. 5:**
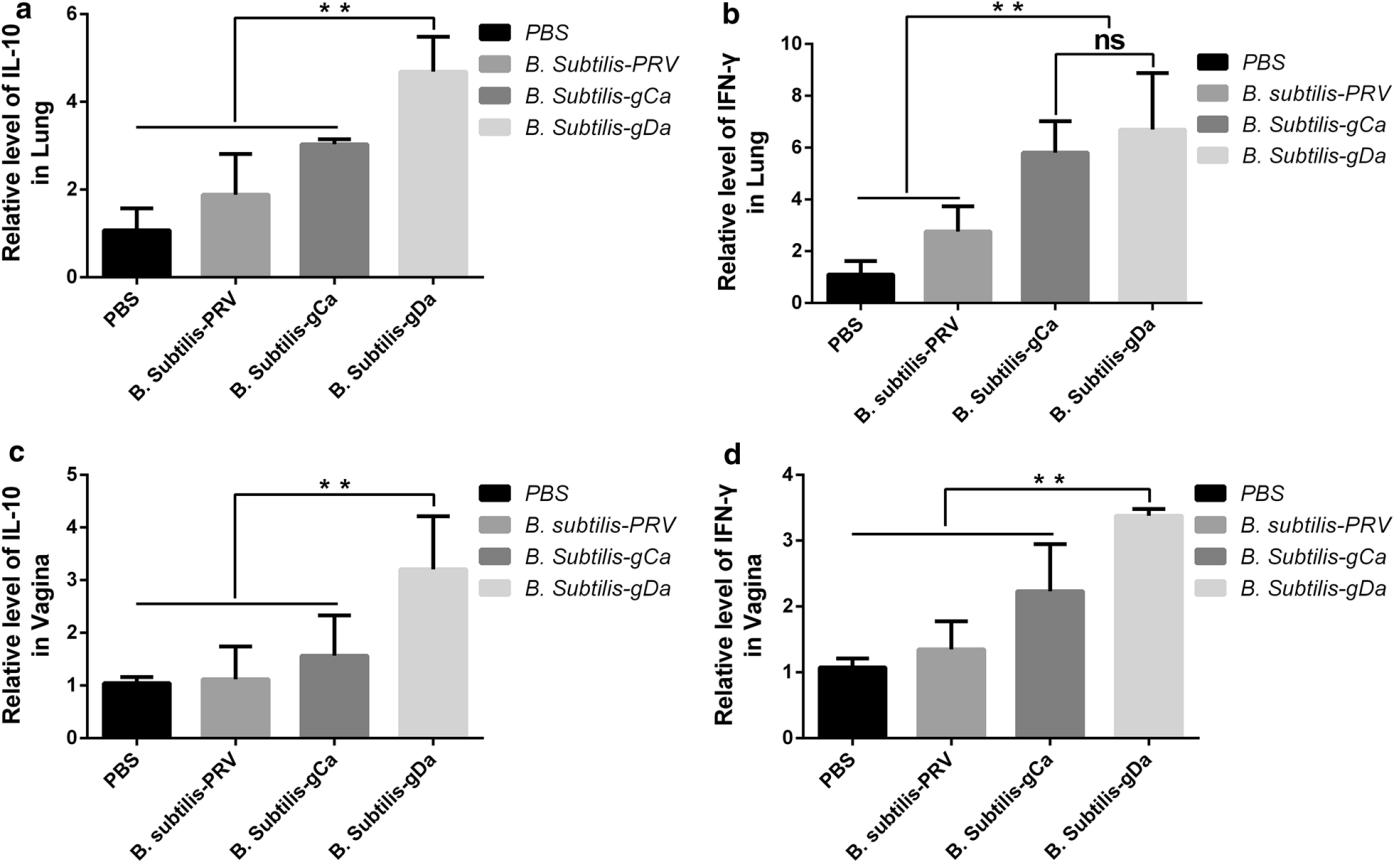
The determination of cytokine responses by RT-qPCR. The mRNA were extracted in lung and vagina. Then, the expression of IFN-γ and IL-10 were detected by RT-qPCR. **a**, **b** Detection of IFN-γ and IL-10 in lung. **c**, **d** Detection of IFN-γ and IL-10 in vagina. Data were expressed as the mean ± SD. **P < 0.01

We tested the ability of recombinant *B. subtilis* to induce production of cytokines. The cytokines IFN-γ and IL-10 were detected by RT-qPCR in lung and vagina. The data showed that intranasal immunization with *B. subtilis*-gDa could effectively stimulate upward IFN-γ and IL-10 in lung (*P *< 0.01) (Fig. 5a, b). However, the IFN-γ had similar expression between the *B. subtilis*-gDa and *B. subtilis*-gCa group (*P* > 0.05) (Fig. 5b). The same cytokine responses were found in vagina (Fig. 5c, d).

### PRV neutralization assay

**Fig. 6:**
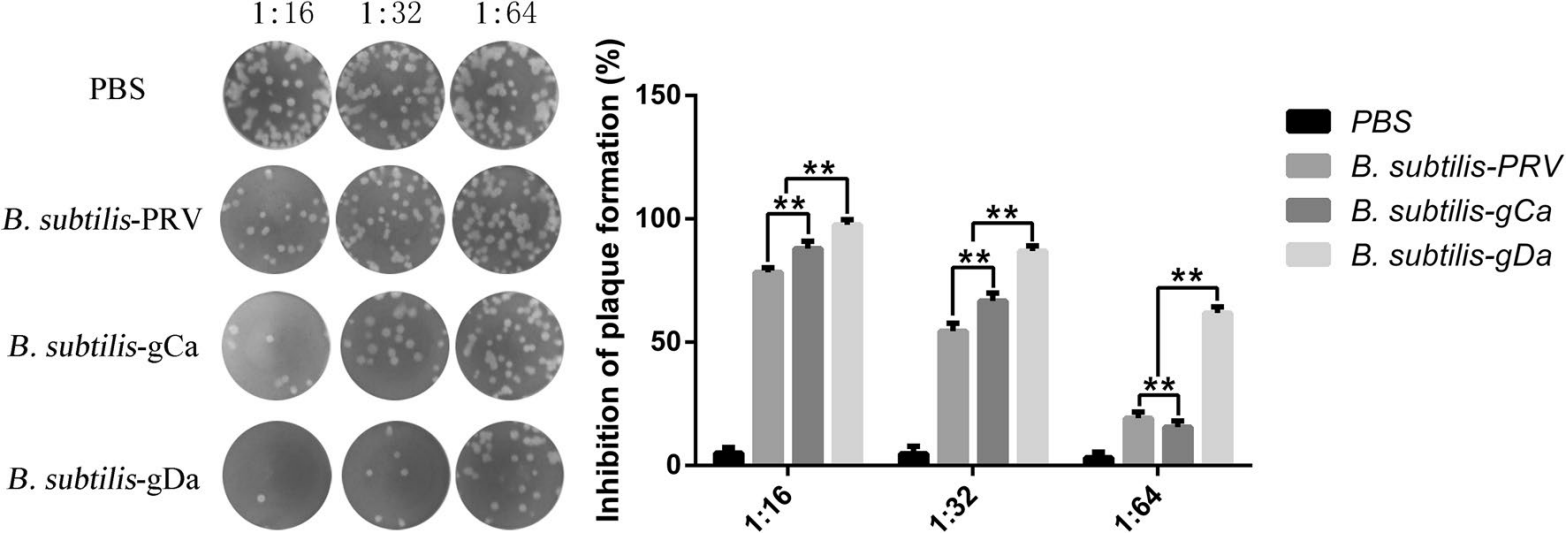
The determination of the PRV neutralizing antibodies. PRV neutralizing antibodies were measured in sera by plaque reduction neutralization test (PRNT). Data were shown as the mean ± SD. **P < 0.01

The neutralizing antibodies from mice intranasal administration with the recombinant *B. subtilis* was evaluated by PRNT (Fig. 6). We observed that *B. subtilis*-gDa had better neutralization than other groups in different dilutions of serum (*P *< 0.01). Besides, *B. subtilis*-gCa significantly induced higher neutralization titer than *B. subtilis*-PRV in dilutions of 1:16 and 1:32 (*P *< 0.01). However, B. subtilis-gCa remarkably declined in dilutions of 1:64 (*P *< 0.01).

## Discussion

*Bacillus subtilis* is an ideal vehicle to carry foreign proteins, and has a full set of biotechnological application. In the present study, we successfully constructed and verified the recombinant *B. subtilis* expressing gCa (*B. subtilis*-gCa) and gDa (*B. subtilis*-gDa). *B. subtilis* possess a predominant advantage in inducing specific immunity [34]. It had been shown that recombinant *B. subtilis* could stimulate humoral and cellular immunity [35]. Therefore, we further explored the immunization efficacy of *B. subtilis*-gCa and *B. subtilis*-gDa.

PRV is one of the most infectious swine diseases and makes great economic losses in the world. PRV genome contains 11 different envelope glycoproteins (named gB, gC, gD, gE, gG, gH, gI, gK, gL, gM, and gN) that interact with host cells [36]. Among them, the gC and gD are the major proteins that elicit immune responses. Recombinant baculovirus which expressed gC and gD had been proved to elicit high titers of PRV-specific serum antibodies of IgG and IFN-γ against PRV challenge [37]. Recombinant DNA vaccines expressing gC and gD had been found to effectively induce either humoral or cellular immunity against PRV infection [38–40]. Besides, through bacterial artificial chromosome (BAC) technology to construct gC and gD substituted pseudorabies virus could produce fantastic relative immune protection [41]. Therefore, using gC and gD proteins to construct recombinant *B. subtilis* is a great choice against PRV infection.

Intranasal administration is a promising alternative to other traditional administration routes, such as oral or parenteral administration. Intranasal administration depends on the large nasal mucosa and induces strong mucosal immunity. Intranasal immunization with CpG oligodeoxynucleotide could significantly increase the titers of specific IgA and IgG antibodies and induce stronger mucosal immune responses than subcutaneous (SC) route [42]. Although intranasal administration is becoming a preferable way of mucosal immunity, in fact, the complex anatomic structure of the nasal cavity and the special physiological function limit its application range. To solve these problems, a number of different techniques have been used to overcome the limitations posed by the nasal mucosa, liking nasal enzyme inhibition, permeation enhancing, drug chemical structure modification and design of pro-drugs and particulate [43]. However, these techniques currently are in the primary phase, and need further research and general application.

In this case, *B. subtilis* shows many advantages. Firstly, *B. subtilis* is able to reach and adhere to the nasal mucosa, more importantly increasing the number of immune cells in the nasal mucosa [44]. Secondly, *B. subtilis* is an acknowledged safe, cheap and well-tolerated probiotics. At last, *B. subtilis* has capacity for genetic manipulation, easy handling, short processing times, and convenience for application in large-scale industrial production [45]. This explains why intranasal administration with recombinant *B. subtilis*-gCa and *B. subtilis*-gDa stimulated a much stronger immunity in mice.

As mentioned earlier, the nasal cavity is a main pathway of PRV infection. Therefore, mucosal immune plays an important role in against PRV infection. Secretory immunoglobulin A (SIgA) is the major antibody isotype in mucosal immune and provides extended protection. Intranasal administration with monoclonal IgA antibodies could significantly suppress the respiratory syncytial virus and prevent initial infection in nasal mucosa [46]. Besides, SIgA antibodies also provides cross-protection against infection with different strains of influenza B virus [47]. Moreover, oral administration with CpG ODNs induces higher titers of IgA against PRV infection [48]. Our data indicated the *B. subtilis*-gCa and *B. subtilis*-gDa could provide sufficiently long-lasting mucosal immunity by increasing the levels of specific IgA in the nasal. In addition, owing to the common mucosal immune system (CMIS), high levels of specific IgA also were detected in vagina, although vagina was so distant from the nasal. The CMIS ensures that lymphocytes induced by an antigen in a mucosal site migrate to other mucosal sites as effector cells to protect all mucosal tissues from the same antigens [49].

Except for mucosal IgA, immunity against PRV depends more on the T lymphocytes. It is demonstrated that the proliferation of CD4^+^CD8^+^ T cells might directly contribute to the elimination of PRV infected cells [50]. Cytokines play critical roles in regulating the antigen-specific T cell responses. T-cell responses are classified as either Th1 (IFN-γ, IL-2) or Th2 (IL-4, IL-10) based on their cytokines, with representing either cell-mediated or antibody-mediated responses, respectively [51]. Administration with PRV vaccine with GSLS-TS significantly elicited robust and unbiased Th1 and Th2 immune responses by up-regulated Th1 (IFN-γ and IL-12) and Th2 (IL-5 and IL-10) cytokines [52]. In addition, intranasal administration with PRV DNA vaccine could induce mixed Th1/Th2-type immune responses and provide protective immunity against PRV infection [53]. Similarly, our results also demonstrated that recombinant *B. subtilis*-gCa and *B. subtilis*-gDa could regulate the specific T lymphocyte proliferative responses, and produced IFN-γ and IL-10 to raise the percentage of CD3^+^, CD3^+^CD4^+^, and CD3^+^CD8^+^ T cells in spleen. The CD3^+^CD4^+^ and CD3^+^CD8^+^ T cells possess the properties of mature antigen-experienced cells and contribute to producing virus-specific antibodies by B cells [54]. Moreover, the CD3^+^CD4^+^ and CD3^+^CD8^+^ T cells could induce a clear PRV-specific CD4-dependent DTH reactivity and a classical CD8-dependent MHC-restricted cytotoxicity [55]. Besides, recombinant B. subtilis-gCa and B. subtilis-gDa could strengthened the systemic immune by upregulating the titers of IgG in serum. The specific virus-neutralizing serum antibodies play a substantial role in controlling PRV infection. Our results showed that recombinant *B. subtilis*-gCa and *B. subtilis*-gDa induced strongly neutralizing antibodies to limit virus replication [56]. These all indicated that *B. subtilis*-gCa and *B. subtilis*-gDa could induce both cell-mediated immunity (CMI) and antibody-mediated immunity (AMI) in protecting against PRV infection.

In conclusion, the present study successfully constructed recombinant *B. subtilis*-gCa and *B. subtilis*-gDa, and demonstrated that intranasal administration with *B. subtilis*-gCa and *B. subtilis*-gDa could effectively stimulate IgA and IgG immune responses, regulate specific T lymphocyte proliferative response by IFN-γ and IL-10, ultimately produce high titers of neutralizing antibodies against PRV infection in mice. In particular, *B. subtilis*-gDa possessed more excellent immune effect. Owing to the outbreaks of PRV in pig farms, *B. subtilis*-gCa and *B. subtilis*-gDa might provide new and key perspectives in developing successful PRV nasal vaccines in swine.

## Additional files

**Additional file 1: Figure S1.** Analysis of the dominant antigen regions. (A) Transmembrane analysis of the whole length of gC protein by TMHMM 2.0 analysis. The results showed that 1–459 amino acids were located outside the membrane, 460–482 amino acids were located on the cell membrane and 483–387 amino acids were located inside the membrane. (B) The whole length of gD protein was analyzed by TMHMM 2.0. The results showed that 1-353 amino acids were located outside the membrane, 354–376 amino acids were located on the cell membrane and 377-402 amino acids were located inside the membrane. (C, D) The secondary structure, hydrophilicity, surface accessibility, flexibility and antigen index of the gC (C) and gD (D) were analyzed by DNAstar Protean software. (E) gC 1417 bp and gD 1139 bp were amplified by PCR. (F) gCa 444 bp and gDa 815 bp were amplified by PCR.

**Additional file 2: Figure S2.** Recombinant fragments cloning. EGFP 717 bp (A), gCa-EGFP 1161 bp (B) and gDa-EGFP 1856 bp (C) were obtained by PCR amplification.

**Additional file 3: Figure S3.** Transformation *Bacillus subtilis* by Electroporation. Electroporation under the conditions of 22 KV/cm, 25 μF, 2000 Ω, 5 ms.
