# Supplementary figures and images for: Intranasal administration with recombinant Bacillus subtilis induces strong mucosal immune responses against pseudorabies
Source: Microb Cell Fact. 2019 Jun 7;18:103. doi: 10.1186/s12934-019-1151-8 (PMC6555017; doi:10.1186/s12934-019-1151-8)

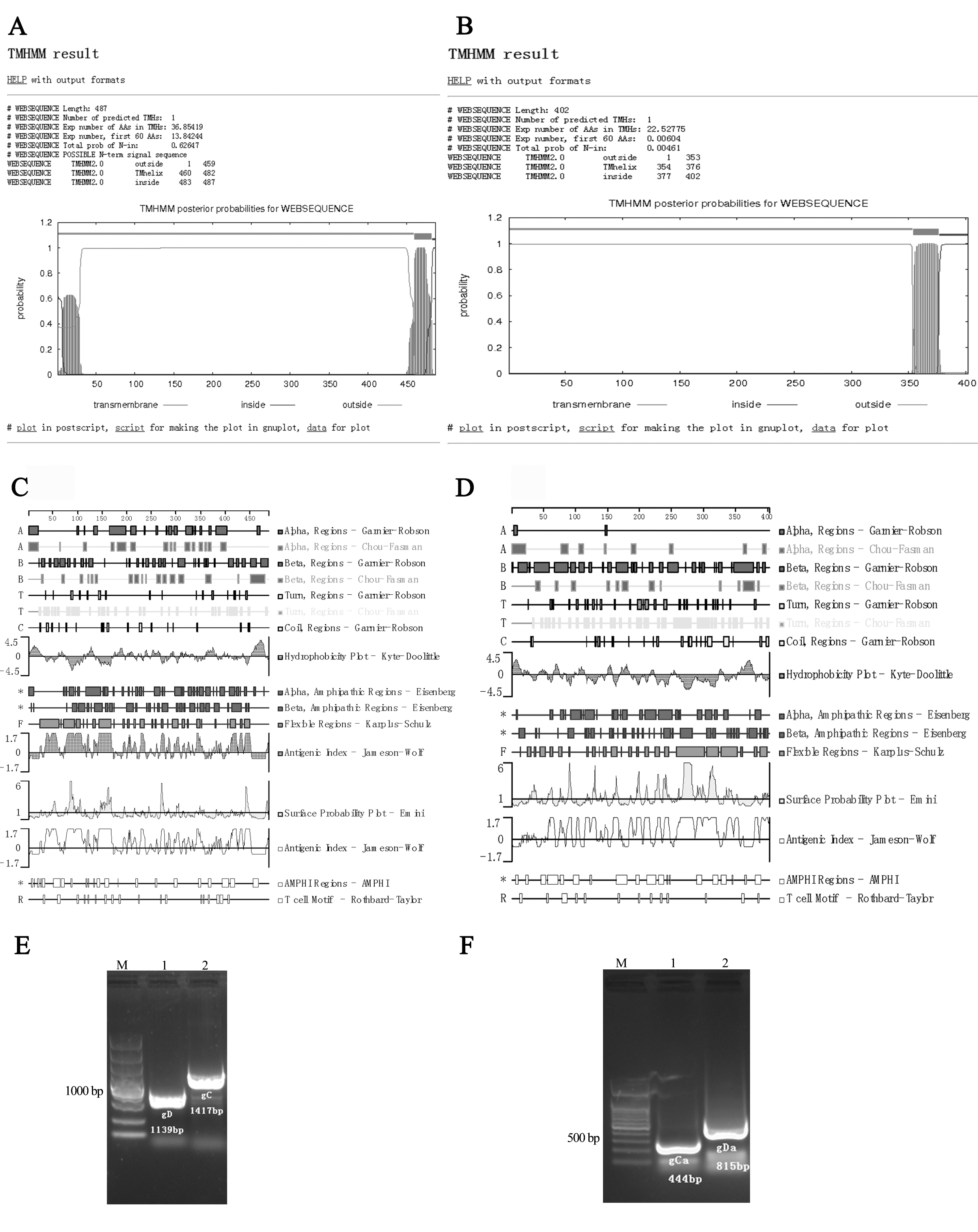

Supplement: Supplementary file 1 — Additional file 1: Figure S1. Analysis of the dominant antigen regions. (A) Transmembrane analysis of the whole length of gC protein by TMHMM 2.0 analysis. The results showed that 1–459 amino acids were located outside the membrane, 460–482 amino acids were located on the cell membrane and 483–387 amino acids were located inside the membrane. (B) The whole length of gD protein was analyzed by TMHMM 2.0. The results showed that 1-353 amino acids were located outside the membrane, 354–376 amino acids were located on the cell membrane and 377-402 amino acids were located inside the membrane. (C, D) The secondary structure, hydrophilicity, surface accessibility, flexibility and antigen index of the gC (C) and gD (D) were analyzed by DNAstar Protean software. (E) gC 1417 bp and gD 1139 bp were amplified by PCR. (F) gCa 444 bp and gDa 815 bp were amplified by PCR. [file 12934_2019_1151_MOESM1_ESM.tif]

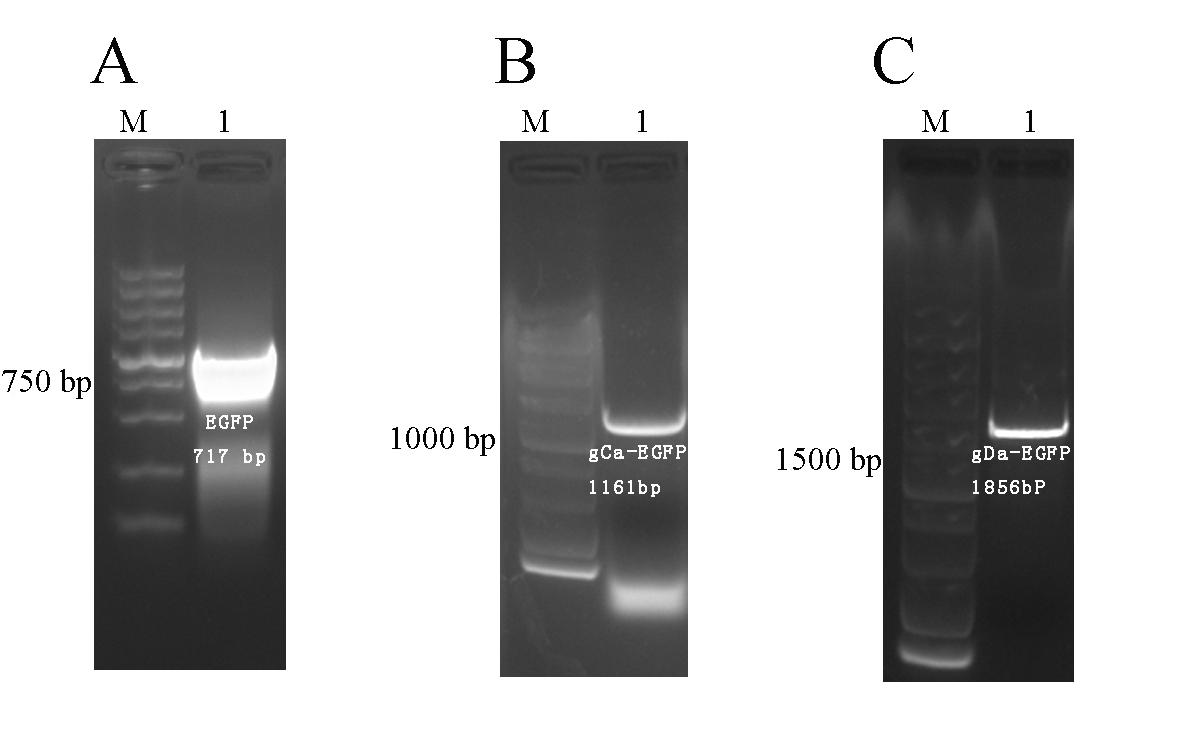

Supplement: Supplementary file 2 — Additional file 2: Figure S2. Recombinant fragments cloning. EGFP 717 bp (A), gCa-EGFP 1161 bp (B) and gDa-EGFP 1856 bp (C) were obtained by PCR amplification. [file 12934_2019_1151_MOESM2_ESM.tif]

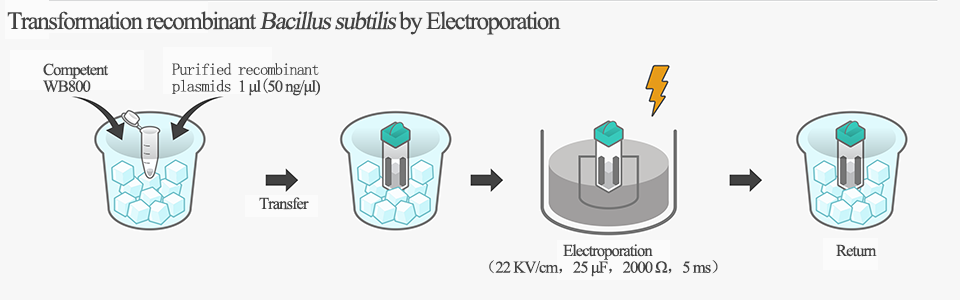

Supplement: Supplementary file 3 — Additional file 3: Figure S3. Transformation Bacillus subtilis by Electroporation. Electroporation under the conditions of 22 KV/cm, 25 μF, 2000 Ω, 5 ms. [file 12934_2019_1151_MOESM3_ESM.tif]
